# Supplementary material for: Identification and functional analysis of non-coding regulatory small RNA FenSr3 in Bacillus amyloliquefaciens LPB-18
Source: PeerJ. 2023 May 15;11:e15236. doi: 10.7717/peerj.15236 (PMC10194069; doi:10.7717/peerj.15236)
Supplement: Supplemental Information 4 [file peerj-11-15236-s004.zip › KO/CK-vs-T1_map/map04145.html]

KEGG PATHWAY: Phagosome - Reference pathway


|  |  |
| --- | --- |
| **Phagosome - Reference pathway** |  |

[
Pathway menu
| Organism menu
| Pathway entry
| Show description
| User data mapping
]

Phagocytosis is the process of taking in relatively large particles by a cell, and is a central mechanism in the tissue remodeling, inflammation, and defense against infectious agents. A phagosome is formed when the specific receptors on the phagocyte surface recognize ligands on the particle surface. After formation, nascent phagosomes progressively acquire digestive characteristics. This maturation of phagosomes involves regulated interaction with the other membrane organelles, including recycling endosomes, late endosomes and lysosomes. The fusion of phagosomes and lysosomes releases toxic products that kill most bacteria and degrade them into fragments. However, some bacteria have strategies to escape the bactericidal mechanisms associated with phagocytosis and survive within host phagocytes.

|  |  |  |
| --- | --- | --- |
| Reference pathway | 184% 150% 122% 100% 82% 67% 55% | 图片下载 |
